# Non-beneficial pediatric research: individual and social interests

**DOI:** 10.1007/s11019-014-9586-5

**Published:** 2014-07-31

**Authors:** Jan Piasecki, Marcin Waligora, Vilius Dranseika

**Affiliations:** 1 Department of Philosophy and Bioethics, Faculty of Health Sciences, Jagiellonian University, Medical College, Michalowskiego 12, 31-126 Kraków, Poland; 2 Department of Logic and History of Philosophy, Vilnius University, 9/1 Universiteto, 01513 Vilnius, Lithuania

**Keywords:** Best interests standard, Children, Non-therapeutic research, Pediatric research, Precedence of individual interests, Research ethics

## Abstract

Biomedical research involving human subjects is an arena of conflicts of interests. One of the most important conflicts is between interests of participants and interests of future patients. Legal regulations and ethical guidelines are instruments designed to help find a fair balance between risks and burdens taken by research subjects and development of knowledge and new treatment. There is an universally accepted ethical principle, which states that it is not ethically allowed to sacrifice individual interests for the sake of society and science. This is the principle of precedence of individual. But there is a problem with how to interpret the principle of precedence of individual in the context of research without prospect of future benefit involving children. There are proposals trying to reconcile non-beneficial research involving children with the concept of the best interests. We assert that this reconciliation is flawed and propose an interpretation of the principle of precedence of individual as follows: not all, but only the most important interests of participants, must be guaranteed; this principle should be interpreted as the secure participant standard. In consequence, the issue of permissible risk ceiling becomes ethically crucial in research with incompetent subjects.

## Background

Biomedical research involving children is still a debated issue. Since the Nuremberg Code, which made voluntary informed consent of a competent participant a necessary condition of medical research, the general approach in national and international policies and ethical guidelines has changed significantly. A number of different aspects of research with children subjects have been discussed extensively. The first step in changing attitudes towards such research was almost universal acceptance of the conviction that participation in biomedical research not only poses risk, but it can sometimes also be beneficial to participants. The next step was the recognition of the importance of evidence based medicine. The main premises here are that only evidence based medicine can be really and consistently beneficial and that no population should become therapeutically orphaned and left without safe and proven therapies (Ross 2004). Consequently, the involvement of children in biomedical research was justified by two important principles of medical ethics: beneficence and justice. References to these principles can be found in many regulations and guidelines. The *Explanatory Report* to the *Convention for the Protection of Human Rights and Dignity of the Human Being with regard to the Application of Biology and Medicine: Convention on Human Rights and Biomedicine*—(Oviedo Convention) explicitly says that banning such research would stop development of science and deprive groups of incompetent people from the benefits of progress in medicine (Council of Europe 1997a). Similar explanation is found in the report issued by the National Commission for the Protection of Human Subjects of Biomedical and Behavioral Research, *Research Involving Children*. This report points out that prohibition of participation of children in biomedical research would be unjust, because the fruits of the progress in medicine would be available to adults but not to children (National Commission 1977). In commentary on CIOMS Guideline 14, it is stated that participation of children is a necessary condition for progress in research into diseases and conditions to which children are susceptible (Council for International Organizations of Medical Sciences 2002). The same reason is given by regulations and recommendations that support research with children. Recent regulations in the US and the EU support research involving children by promising substantial incentives for the pharmaceutical industry. In the EU, pharmaceutical companies are obliged to present a *Paediatric Investigational Plan*, which is thought to influence research and development of medicinal products for pediatric use (European Commission 2006). These regulations are deemed to stimulate pharmaceutical industry. European agencies are becoming involved in the process of development of new treatments for children. Recently, the European Medicines Agency issued a 5-year Report to the European Commission that shows the results of implemented Pediatric Regulation (European Medicines Agency, Paediatric Committee 2012). Also the European Commission published a report to the European Parliament and the European Council. This is a report on experience acquired as a result of the application of Paediatric Regulation, and it shows that development of pediatric drugs has become one of the important issues of European politics (European Commission 2013). Although there are common regulations within the European Union, there are still important differences between laws of particular member-states. The Clinical Trials Directive does not determine all aspects of research with minors. For instance, it does not provide the definition of ‘minor’. Besides that its scope is limited to the area of clinical trials. Therefore, the Directive does not regulate other kinds of medical and other research involving children (European Parliament, Council of the European Union 2001). Thus, in some EU members’ research, which are not clinical trials, are not regulated by legislation, for instance in Ireland (Sheikh 2008). In others, like in Belgium, all types of research is regulated by the law (Pinxten et al. 2008). Another issue is that some EU members follow solely the Directive. Other EU members either ratified or implemented standards proposed by the Oviedo Convention and its protocols (Gevers 2008). Oviedo Convention is a document issued by the Council of Europe, an organization of all European and some non-European countries that aims at cooperation and promoting democracy and human rights. These two documents, namely the Directive and Oviedo Convention, differ in some important aspects, for instance in risk–benefit ratio and in respecting minor’s dissent. Moreover, the systems of ethical assessment of research widely differ in all states of the EU (Kenter and Cohen 2012). The Directive was deemed to be the cause of the decrease in clinical trials in the EU, and increase in their costs. In 2016 it will be replaced by the new Regulation (European Commission 2012). But the new Regulation will not significantly change the situation of research with minors and incapacitated patients. Also it will not unify the system of ethical assessment. Some even expressed a regret that the Regulation will not unify the system and might even contribute to decrease of its quality (Heringa and Dute 2013; Waligora 2013; Westra et al. 2014).

Empirical research conducted in some European countries shows law in some cases might be flexibly applied. For instance, in the Netherlands promising research without prospect of direct benefit, posing more than minimal risk, was in some cases considered acceptable (Westra et al. 2010). However Dutch law allows non-beneficial research with incompetent subjects only, when it posed negligible risk and minimal burdens (Westra et al. 2010; Kenter 2008). Also in Germany, which seems to be one of the most conservative countries in the EU in regards to non-beneficial research, Research Ethics Committees (RECs) chair-persons significantly vary in their attitudes towards non-therapeutic research (Lenk et al. 2004). Also in the US there are doubts about some aspects involving incompetent subjects in research. There is still discussion on what counts as direct and indirect benefits of research (Friedman et al. 2012; Joffe et al. 2006), what is a proper definition of research (Kass et al. 2013), and what limits of risk could be acceptable in non-beneficial research (Wendler 2013).

## The precedence of the individual in regulations

The principle of precedence of individual interest has different formulations in many international regulations. All versions of the Declaration of Helsinki quote phrases from the Declaration of Geneva, saying that “The health of my patient will be my first consideration” and the International Code of Medical Ethics claiming that “A physician shall act in the patient’s best interest when providing medical care” (World Medical Association 2008; 1964; 1996; 2013; 2000). The 1968 version did not go further than these introductory statements. In the 1975 version, Paragraph 5 of “Basic principles” declares that “Concern for the interests of the subject must always prevail over the interest of science and society” (World Medical Association 1975). In Section 3 of that version of the Declaration, devoted to non-therapeutic biomedical research, Paragraph 4 states “In research on man, the interest of science and society should never take precedence over considerations related to the well-being of the subject” (World Medical Association 1975). Subsequent versions have the same wording; new formulations appear in the 2000 and 2008 versions (World Medical Association 2000; 2008). Finally, the current version of the 2013 Helsinki Declaration states “While the primary purpose of medical research is to generate new knowledge, this goal can never take precedence over the rights and interests of individual subjects” (World Medical Association 2013).

The second Article of the Oviedo Convention explicitly states that “the interests and welfare of the human being shall prevail over the sole interest of society or science” (Council of Europe 1997a). The Explanatory Report to the Oviedo Convention explains that in conflict of interests in principle, the interests of the individual must take precedence over the “sole” interests of science and society. The Additional protocol concerning biomedical research and its Explanatory Report contains the same provisions and clarifications. Generally, the Convention is “inspired by the principle of the primacy of the human being” (Council of Europe 1997b). Article 17 of the Convention states that non-beneficial research involving incompetent persons can be conducted in exceptional circumstances. However, non-beneficial research is thought to pose a serious risk of instrumentalization/exploitation of individuals and vulnerable groups. There is an inner tension between different provisions of the Oviedo Convention. The authors of the Convention realized that non-beneficial research might infringe upon interests of incompetent participants. Such research is inconsistent with the principle of the primacy of the human being. Nevertheless, they decided not to ban it. Non-beneficial research is allowed, but only in exceptional circumstances and “on certain strict conditions”. However, in Germany, for instance, acceptability of this research in the Convention was a reason not to ratify it (Stuhlinger et al. 2009; de Wachter 1997) (Table 1).

**Table 1:** Formulations of the principle of precedence of individual interests

Ethical guideline	Year	Formulation
Oviedo Convention	1998	Article 2 “The interests and welfare of the human being shall prevail over the sole interest of society or science”
Directive 2001/20/EC	2001	Article 4, par. i, and Article 5, par. h “The interests of the patient always prevail over those of science and society”
Universal Declaration on Bioethics and Human Rights	2005	Article 3, par. 2. “The interests and welfare of the individual should have priority over the sole interests of science or society”
Ethical Considerations for Clinical Trials on Medicinal Products Conducted with the Paediatric Population	2006	(11) “The child’s interest should always prevail over that of science and society. This is paramount when assessing and monitoring risk. Risk is to be viewed in balance to the benefit”
Regulation of the European Parliament and of the Council on clinical trials on medicinal products for human use, and repealing the Directive 2001/20/EC	2012	Article 28, par. 2. “The rights, safety and well-being of the subjects shall prevail over the interests of science and society”
Declaration of Helsinki	2013	Section I, Paragraph 8 “While the primary purpose of medical research is to generate new knowledge this goal *can never take precedence* of the rights and interests of individual subjects”

A similar wording to the Oviedo Convention can be found in the Ethical Considerations for Clinical Trials on Medicinal Products conducted with the Paediatric Population. These were published by an Ad hoc group for the development of implementing guidelines for Directive 2001/20/EC. Section 11 states: “The child’s interest should always prevail over that of science and society. This is paramount when assessing and monitoring risk. Risk is to be viewed in balance to the benefit” (Ad hoc committee 2006). The Directive states: “the interests of the patient always prevail over those of science and society.” It is interesting to note that this principle appears in the Directive twice in the context of research involving children (Article 4, par. i) and research with incapacitated adults not able to give informed legal consent (Article 5, par. h). The new Regulation moves the principle of precedence to the article (Article 29) containing the general rules. CIOMS Guidelines do not explicitly contain the principle of precedence of the individual, although this requirement is referred to at least twice in the commentaries. Firstly, it is mentioned in the commentary to Guideline 8 that concerns benefits and risk of participation in research, where the Declaration of Helsinki is quoted. Next, it is invoked in the commentary to Guideline 14, which states that a parent might withdraw his or her child from participation in clinical trials when it is not in the child’s best interests (Council for International Organizations of Medical Sciences 2002). Also the *Universal Declaration on Bioethics and Human Rights* issued by UNESCO contains the principle of precedence of individual (Article 3, par. 2) worded as follows: “The interests and welfare of the individual should have priority over the sole interests of science or society”. The *Universal Declaration* seems to reconcile non-beneficial research with incompetent persons with the best interests of participants. Article 7, point a reads “authorization for research and medical practice should be obtained in accordance with the best interest of the person concerned” and in point b states that “research should only be carried out for his or her direct health benefit, subject to the authorization and the protective conditions prescribed by law, and if there is no research alternative of comparable effectiveness with research participants able to consent. Research which does not have potential direct health benefit should only be undertaken by way of exception, with the utmost restraint, exposing the person only to a minimal risk and minimal burden and if the research is expected to contribute to the health benefit of other persons in the same category, subject to the conditions prescribed by law and compatible with the protection of the individual’s human rights” (UNESCO 2005).

## Research involving children and exploitation

The principle of precedence of individual interests can be interpreted in two ways. First, the strong interpretation would bind this principle with the best interests standard. The weaker interpretation would imply only the secure participant standard. The term “the secure participant standard” is an adapted version of “the secure child standard” proposed recently by Sarah Shah (2013). According to the strong interpretation, research is unethical if it is not in the participant’s best interests. The weak interpretation says only that research should not expose a participant to undue, serious risk. The latter proposal sets a certain ceiling of risk, the former sets a requirement of either positive or at least neutral balance of risks and benefits for the individual participant. Therefore, it seems clear why research without prospects of benefit becomes a focal point. If non-beneficial research with children might be reconciled with the best interests standard, the precedence of the individual interests principle can be understood in accordance with the strong interpretation. However, if non-beneficial research might not be reconciled with the best interests standard, it means that if we still want to agree that such research can be acceptable, the principle should be interpreted in the weaker way.

Some argue that in non-beneficial research incompetent patients are used, instrumentalized or even exploited (Johansson and Brostrom 2012). Although one may argue that if non-beneficial research associated with minimal risk brings some benefits to peers of participants, this is not a case of exploitation. But as Johansson and Brostrom realize this is not the case when one group exploits another group. Rather this is a case when one group (future pediatric patients) takes advantage of individuals who do not benefit from research, although they face risk and bear the burden of participation. If non-beneficial research cannot be reconciled with the best interests standard, it would be justified to think that participants are exploited. However it does not mean that some degree of exploitation cannot be ethically justified in some cases.

Usually exploitation is defined as *taking unfair advantage of someone for one’s benefit* (Resnik 2003; Macklin 2004; Wertheimer 1996). Unfair transaction between A and B might occur, when A harms, or disrespects or acts unjustly toward B (Resnik 2003). Exploitation has different degrees and in some cases a certain degree of exploitation might be morally justified (Resnik 2003). For instance we justify some degree of exploitation in the free market to promote social good. We accept that partners may agree to unjust shares in a company. It seems that also non-beneficial research involving children is this kind of justifiable exploitation. Resnik claims that in the case of non-beneficial research, the key issue is the level of harm to a participant that can be justified by the benefits to society (Resnik 2003). If non-beneficial research cannot be reconciled with the best interests standard, the crucial ethical question is an acceptable risk ceiling. Therefore discussion about protection of incompetent subjects should focus on this problem. Moreover as Johansson and Brostrom point out (Johansson and Brostrom 2012), we should be aware that we exploit incompetent subjects in non-beneficial research. This fact should not be hidden by idealistic language. This is because only by being aware of this fact we can properly assess balance between risk and burden and expected benefits for incompetents as a group.

## Regulations on minimal risk

For purposes of argument, we assume that non-beneficial research with incompetent subjects is morally justified and needed. Non-beneficial research is also approved in quoted regulations, although there is not one standard of risk. For instance the Oviedo Convention requires that non-beneficial research be associated only with minimal risk and burden. According to the Additional Protocol, risk and burden cannot be increased, even if research might promise increase of some additional benefit. Risk and burden are understood in a nonrelative way (Article 17). Risk is considered to be minimal when “having regard to the nature and scale of the intervention, it is to be expected that it will result, at most, in a very slight and temporary negative impact on the health of the person concerned.” Minimal burden is defined as temporary and very slight discomfort (Council of Europe 2005). The Declaration of Helsinki also requires minimal risk and burden (Article 28), but does not provide interpretation of this concept (World Medical Association 2013). The Directive as well as the new Regulation do not distinguish between beneficial and non-beneficial research. They both require benefit for the group and minimizing risk and burden “in relation to the disease and developmental stage” (Article 4, par. g; Article 30, par. g) (European Parliament, Council of the European Union 2001; European Commission 2012). But the new Regulation limits the risk and burden of research to the minimal in clinical trials in emergency situations (Article 32, par. e) (European Commission 2012). These concepts are not defined. Ethical considerations (12.1) in non-beneficial research allows for minimal risk or minor increase over minimal risk, when research is associated with benefit for the group and benefit-risk balance is “at least as favorable as that of available alternative approaches” (Ad hoc committee 2006). Minimal risk is defined as risk not greater than encountered in daily life or during performance of physical or psychological test. Appendix to the Ethical considerations gives examples of such defined risk and burden. The UNESCO *Universal Declaration* allows only minimal risk and minimal burden, but does not provide either definition of these categories or examples of procedures.

This overview shows that although all regulations refer to the principle of the precedence of individual, they interpret it differently. According to the Directive and the new Regulation, the principle can be reconciled with minimizing risk and burden. According to the *Oviedo Convention*, *Universal Declaration*, CIOMS *Guidelines* and *Declaration of Helsinki,* the principle of precedence of individual can be reconciled with research posing minimal risk and burden. Finally, according to *Ethical considerations,* this principle can be reconciled with research posing minor increase over minimal risk. Also only the *Oviedo Convention* and *Ethical considerations* give quite clear interpretation of risk. It seems that at least in the EU some compromise and unified standard is possible.

## The principle of precedence of individual interests and the best interests standard

The best interests standard in the context of health was an object of criticism both in medical and legal contexts. It was argued that this is an open-ended, indeterminate and speculative concept. Also, it was accused of being vague, unknowable and unrealistic. Other arguments indicate that the best interests standard is not a helpful tool, because if one wants to resolve a conflict of interests between many different parties, it does not answer whose best interests should be considered (Archard 2013; Elliston 2007). Recently, nevertheless, the best interests standard received strong support from Loretta Kopelman (1997a, b, 2002, 2007, 2012) and there were some attempts to reconcile this concept with non-beneficial research (Litton 2008). We do not want to discuss or revise the best interest standard, as Kopelman does it—the main issue here is to formulate the proper interpretation of the principle of precedence of individual that can be reconciled with research without prospect of future benefit involving incompetent subjects. For that purpose, the two different standards are distinguished. Both the best interest standard and the secure participant standard determine different limits of moral obligation towards children. According to the best interest standard, health interests of incompetent persons cannot be sacrificed for the purpose of research in interests of other parties. In other words, according to the best interests standard, a child might participate in biomedical research only when it does not endanger her interests. The secure participant standard allows for compromising such child’s interests, but only to a certain point—for instance within the limits of minimal risk or minor increase over minimal risk.

## Proper understanding of the best interest standard

If a research participant gives her informed consent for participation, she is thought to act in her own best interest. Her interest is understood here more broadly than only in medical terms. Beliefs and preferences of the autonomous person are the grounds constituting her best interests (Berger 2011). But in the case of non-beneficial research involving incompetent subjects, an important question arises. The best interests of a non-autonomous person are usually defined in medical terms (Berger 2011). It does not mean that opinions and beliefs of incompetent persons do not matter. On the contrary, they have significance. It is universally recognized that a researcher should seek the incompetent participant’s assent and respect her dissent (Waligora et al. 2014). Participation needs authorization of a legal representative. Participation in non-beneficial research brings no clinical benefit for the subjects involved, but it still is normally associated with non-zero risk and burden. However, there are some proposals how to reconcile participation in non-beneficial research with the best interests standard.

L. M. Kopelman gives an interesting analysis using the best interests standard in policy and decision-making involving incompetent persons (Kopelman 2007, 1997a). According to Kopelman, the best interests standard can be understood in a threefold manner. Firstly, it can be conceived as a threshold for intervention and judgment. In this case, the best interests standard consists in establishing the minimum acceptable standard of care that must be fulfilled. In the case when parents’ decisions endanger the well-being of a child, a court might try to find the most reasonable way of action in those circumstances. Secondly, the best interests standard can be understood as an ideal that directs guardians and policy makers in decision-making processes towards promoting the wellbeing of those incapacitated and establishing prima facie duties to them. In this sense, the best interests standard might be helpful in clarifying duties and in balancing different interests. Finally, the best interests standard might be understood as a standard of reasonableness. As such, it can also be used in custody decisions and medical decisions. It does not imply that others’ interests can be neglected, but it helps to find the best solution in a certain situation: the best solution for a child’s well-being in the physical and psychological sense. As Kopelman writes, the best interests standard in this, third, sense might be considered as instruction to choose that option that would be chosen by the most informed rational person of good will. This option would maximize the child’s benefits and minimize child’s harm and at the same time would also take into consideration the rights, needs and interests of other people. This analysis spreads the sense that is usually attached to the concept of the best interests standards. The first concept analyzed by Kopelman should be rather called a *minimal interest* *standard* and it is probably the closest concept to the secure participant standard. This standard does not refer to what exactly is a child’s due, but rather to what extent the child’s needs and interest can be compromised. Moreover this concept is derived from the second understanding of the best interests given by Kopelman. One has to establish the child’s needs and interests in the ideal situation, and then try to determine to what extent these needs and interest can be compromised. Kopelman prefers to use the term *the best interest standard* probably to be in accordance with legal tradition and terminology, not because of the content of the concept itself.

## Reconciliation between the best interests standard and non-beneficial research?

Kopelman also draws a line of possible argumentation, pointing out that the policy introducing the best interests standard as a threshold for parental authority is more beneficial for all children than a lack of such a policy. This line of argumentation might be developed to reconcile non-beneficial research and the best interests standard. A policy that allows for non-beneficial research with children is generally more beneficial for every individual child than a policy banning such research. In society, where a policy allowing non-beneficial research exists, every child is better off, because she benefits from scientific development more than a child in a society where benefits of development of medicine are mainly confined to the adult population. Litton is convinced that his statement to the effect that the best interests standard is reconcilable with the policy for allowing non-beneficial research has empirical character (Litton 2008).

This argument assumes that not all regulations that allow non-beneficial research involving children would be beneficial to every child, even those who are participating in studies that expose them to some level of risk. For example, it might seem that a policy that sets a ceiling of risk for non-beneficial research would be more beneficial than a policy which does not set any limit of risk. A policy that does not set a risk ceiling might be in accordance with utilitarian calculus beneficial for the whole population, but not beneficial for participants who would be exposed to very high risks. Following this line of argumentation, such policy should be rejected and a policy that sets a certain risk ceiling established—the policy which is beneficial for both every individual child and children as a group—it is debatable what exactly this risk ceiling should be.

It is sometimes argued that non-beneficial research involving subjects who cannot give informed consent entails exploitation of participants. A participating individual, if she does not benefit from the research, is treated merely as a means to produce good for others. However, Litton’s approach points at a broader context of biomedical research and thereby allows to invert this argument. If a policy allowing non-beneficial research is beneficial to every individual child, a principled refusal to let a child participate in research rather entails treating other children merely as means towards one’s ends (Litton 2008). It implies that if the policy allowing research is beneficial to both all children and every participant in particular, a person, who does not want to follow the policy without sufficient reason and in principle refuses to be involved in research, is a free-rider, who benefits from the fact that others do participate. Therefore, he or she treats others only as means to his or her own goals.

This argument nevertheless is fragile to objections (Shah 2013). There are children who remain relatively healthy throughout their childhood and who do not need new therapies. For them, the policy allowing non-beneficial research is not beneficial. Moreover, this policy may expose them to risk that is greater than they would be exposed to if there were no such policy. Besides that, some children are likely to be exposed to a very small risk of death as a consequence of being involved in biomedical research. Sarah Shah argues that the risk of death as a consequence of participation in medical research is similar to the risks associated with being treated for a disease that child might contract. That argument would be difficult to make after the child’s death (Shah 2013). Summing up, we state that Litton has successfully argued that it is not fair to principally refuse participation in activity that brings one some profits even if it might be associated with some risk. Nevertheless, this argument does not reconcile the individual’s best interests with non-beneficial research.

## The objective good and the best interests

There is an alternative to reconciling the best interests standard with acceptability of non-beneficial research. According to David Wendler, some individuals might later in life finally come to understand and embrace their contribution to the development of science when they were children and that this can become a part of their biography and make their lives better. Therefore, it can be concluded that involvement in non-beneficial research can promote individual interests (Wendler 2012). Wendler’s argument hinges on two premises. The first is that enrolling a child into a clinical trial might be consistent with her clinical interests *ex ante* (before the event), by which he means that a study eventually can turn out to be harmful or non-beneficial, but before its launch it could present acceptable risk/benefit ratio. The second says that individual interests can be understood not only in terms of pleasure, and satisfaction of preferences, but also objectively. It means that something can influence one’s life, making it better or worse, even though it has nothing to do with pleasures or current preferences. To illustrate this kind of good, Wendler gives an example of a 2-year-old child accidentally and innocently killing a friend. Such event—even if it does not affect a child in terms of pain or compromising preferences, may nevertheless influence her life and by becoming a part of one’s life’s narrative, would make it worse. Then Wendler argues that involvement in non-beneficial research might be embraced by a formerly incompetent participant as important, but also it might make her life better in the objective sense, even if one does not even think that one has done something objectively praiseful.

Wendler tries to refute possible objections and he claims that even if it turns out that a former participant rejected and dismissed her participation and contribution, it would not mean that risk/benefit ratio of participation had been unfair *ex ante* (before the event). Kopelman aptly realizes that, at this point, there are no data indicating how many former subjects benefit in a way described by Wendler (Kopelman 2012). These data are needed to estimate the exact risk/benefit ratio. One can say that Wendler has in mind the objectively understood interest of an individual and it does not matter, if one is capable to embrace it or not. Therefore, Kopleman’s demand for research would be a misunderstanding. But the very concept of the objective interests is quite controversial. One can take a stand that there is nothing like objective value. All values are relational entities. Therefore, it is not true that a participant’s life is better, even when she dismisses her involvement. In this situation, there are only some people who perceive her life as more meaningful and better. Thus it can be said that the participant’s recognition of her involvement in medical research is crucial in regards to her best interests. And, as a consequence, the argument does not extend to include all incompetent subjects. For example, it does not work for persons permanently incapacitated by dementia who did not leave any testimonies expressing their preferences concerning participation in research. The same might be said about young children who, due to serious diseases, never reach a certain stage of cognitive development. For these people, there will not be a moment in which they relate themselves to the research in which they participated. Therefore, participation in its moral sense would not be in their interest. In addition, their best interest should be defined rather in biological than in moral terms. Moreover, as Linus Broström and Mats Johansson point out, this argument relies on the *mere* possibility of benefiting from research (Brostrom and Johansson 2014).

## The secure participant standard

Kopelman gives the following example. Parents want to enroll their 3-year-old child in an endocrinology trial (Kopelman 2007). The trial is not meant to bring any direct benefit to the child and requires a 2-day stay in a hospital and several injections. The parents’ decision of involvement of their child into this study, according to Kopelman, would not meet the best interests standard understood as a threshold—parents would not ensure their child’s safety and they would expose her to risk that is too high. However, society allows parents to engage their children in many risky activities, for instance sports (football, karate and skiing). It is assumed that parents usually take care of children’s interests, but parental authority is not a logical derivative from the concept of the child’s best interests (Downie and Randall 1997). Parenting is a value in itself and parental powers stem from the rights to privacy and intimate relationships (Downie and Randall 1997). These rights can be limited only if parents endanger the well-being of their child in a socially unacceptable manner. In this example, although Kopelman invokes the concept of the best interests, she conceives it as a limitation of risks. Parents are not entitled to put their children at too serious a risk. But this formulation should be called rather a secure participant standard instead of the best interests standard, because parental decision in this case is not considered unacceptable, simply because it does not promote the child’s best interests. It was probably even not intended to promote them. It exposed a child to undue risk.

The “secure child standard” was proposed by Sarah Shah (2013). This standard might be nevertheless extended to all incompetent research participants. The secure participant standard allows the incompetent to be involved in different non-zero risk procedures, if the risk is justified. The best interests standard is especially difficult to apply in the context of biomedical research, because the main goal of research is to produce generalizable knowledge, not individual benefits and it is not meant to promote individual interests. In non-beneficial research involving incompetent subjects, individual risk and discomfort is weighed against development of science and the prospect of benefits for the future patients. The main concern, therefore, is not the promoting of the participants’ well-being, but rather checking if her well-being is not endangered too much. The principle of the precedence of individual interest has, therefore, one specific task: to protect a participant from undue risk. The principle of precedence of the individual was established to stop exclusively utilitarian logic, and in this sense it has deontological elements. This principle is used to stop aggregation of the rights and interests of society and science to outweigh important interests and rights of individual participants (Pattinson 2012). The deontological approach does not inevitably entail prohibition of balancing different interests, and, in particular, weighing interests of the individual against interests of society. If participation in biomedical research does not endanger significant interests of subjects, then it can be considered justified exploitation. Then the main task here will be to set up a limit of risk that a participant can be exposed to without being unjustifiably exploited. It seems there is still not a compromise in the EU about risk requirements in non-beneficial research involving incompetent subjects.

## Conclusion

We propose that there is no sufficient argument yet that proves non-beneficial research can ever be in the best interests of incompetent participants. It is rather otherwise: in non-beneficial research with non-zero risks, important interests of incompetent subjects are sacrificed for the good of others and science (Johansson and Brostrom 2012; Brostrom and Johansson 2014). Ethical guidelines and regulations should not veil this very fact and cover it with hypocritical language. Hypocrisy might be understood as lack of moral seriousness and significant demand for ethical documents is to be morally serious (Crisp and Cowton 1994). Moreover, the strong interpretation of the principle of the precedence of the individual would be inconsistent with the part of regulations that allows non-beneficial research to be conducted with children. Even if we accept that it is sometimes morally acceptable to balance interests of children and interests of society and future patients, it would be wrong to apply unrestricted utilitarian calculus that can sometimes lead to unacceptable sacrifice of the individual’s rights and interests for the sake of well-being of the society. For this reason, regulations concerning biomedical research with children should protect their interests and should not allow for exclusively utilitarian calculations. In general terms, this limit could be expressed as the principle of precedence of the individual (in proposed weak interpretation) and it should take a form of requirement of risk ceiling as a specific and operational provision.
